# Association Between Maternal C-Reactive Protein (CRP) Levels and Adverse Neonatal Outcomes: A Systematic Review and Meta-Analysis

**DOI:** 10.3390/jcm15062114

**Published:** 2026-03-10

**Authors:** Rutaba Mahereen, Abdullah Alsatli, Faiza Said Albader, Rawan Ibrahim Alqabbaa, Lamar Abu Shehadeh, Mohamad Behairy, Ghezlan Alaliw, Lamees Tarek Alzahrani, Maria Abdulaziz Alrafi, Nojoud Sulaiman Alganas, Noor A Altaho, Saeed Baradwan, Ayman MA Mohamed, Ahmed Abu-Zaid

**Affiliations:** 1 College of Medicine, Alfaisal University, Riyadh 11533, Saudi Arabia; 2 College of Medicine, Almaarefa University, Riyadh 13713, Saudi Arabia; 3 Department of Pediatrics, Al-Amiri Hospital, Kuwait City 13001, Kuwait; 4 Department of Obstetrics and Gynecology, King Faisal Specialist Hospital and Research Center, Jeddah 23433, Saudi Arabia

**Keywords:** C-reactive protein, maternal inflammation, preterm birth, low birth weight, neonatal outcomes, pregnancy biomarkers, stillbirth, small for gestational age

## Abstract

**Background:** C-reactive protein (CRP), a biomarker of systemic inflammation, has been implicated in adverse pregnancy and neonatal outcomes. However, the relationship between maternal CRP and neonatal complications remains unclear. We conducted a systematic review and meta-analysis to synthesize available evidence. **Methods:** We systematically searched PubMed, Scopus, Web of Science, and Cochrane Library up to July 2025 for observational studies reporting maternal CRP levels in relation to neonatal outcomes. Eligible outcomes included preterm birth (PTB), low birth weight (LBW), small for gestational age (SGA), and stillbirth. Random-effects models were used to calculate pooled standardized mean differences (SMD) or odds ratios (OR) with 95% confidence intervals (CI). Statistical heterogeneity was assessed using the I^2^ statistic. **Results:** The search yielded 6843 records, of which 42 studies (comprising 18,393 pregnant women) met the inclusion criteria. Maternal mean CRP levels were significantly higher in adverse pregnancy outcomes compared with controls (SMD = 0.39; 95% CI 0.08–0.70; *p* = 0.01; I^2^ = 96.6%). Elevated CRP was strongly associated with PTB (OR = 3.81; 95% CI 2.66–5.47; *p* < 0.001; I^2^ = 85%; *n* = 23) and LBW (OR = 2.34; 95% CI 1.35–4.03; *p* = 0.002; I^2^ = 84.2%; *n* = 7). No significant associations were observed for SGA (OR = 1.14; 95% CI 0.86–1.49; *p* = 0.36; I^2^ = 0%; *n* = 5) or stillbirth (OR = 1.89; 95% CI 0.92–3.90; *p* = 0.08; I^2^ = 44.9%; *n* = 4). **Conclusion:** Maternal CRP is significantly associated with increased risks of preterm birth and low birth weight but not with SGA or stillbirth. These findings support the role of systemic inflammation in adverse neonatal outcomes and highlight the need for prospective studies to clarify causal mechanisms and assess the clinical utility of CRP in pregnancy risk stratification.

## 1. Introduction

Pregnancy is a highly complex physiological process that requires the balanced adaptation of maternal, placental, and fetal systems to ensure healthy development [1,2]. Despite significant advances in obstetric and neonatal care, neonatal complications remain a major contributor to global morbidity and mortality [3,4]. Each year, an estimated 13.4 million infants are born preterm (before 37 weeks of gestation), accounting for nearly 1 in 10 live births worldwide [5]. Furthermore, 20.5 million babies are born with a low birth weight (LBW, <2500 g) [6,7]. Preterm birth (PTB) complications are the leading cause of death in children under 5 years of age, responsible for approximately 1 million neonatal deaths annually [8,9]. LBW significantly increases a newborn’s vulnerability, elevating the risk of infections, impaired neurodevelopment, and chronic diseases such as diabetes and hypertension later in life [10,11,12]. These challenges are compounded by other conditions; being small for gestational age (SGA) affects an estimated 27% of live births (nearly one in four newborns globally) [13,14].

It has been demonstrated that systemic and chronic inflammation is increasingly recognized as a key pathway in adverse pregnancy and neonatal complications [15,16,17]. During a normal pregnancy period, the maternal immune system balances tolerance to the fetus by increasing its protection against pathogens and inflammation [18]. When this balance is disrupted, increased inflammatory responses can compromise placental function and fetal growth [19]. C-reactive protein (CRP), an acute-phase protein synthesized by the liver in response to interleukin-6 and other cytokines, is a widely used marker of systemic inflammation [20,21]. In pregnancy, higher CRP levels have been observed in association with infections, gestational disorders, and placental pathology, making it a biologically plausible marker for adverse neonatal outcomes [22,23,24].

Several observational studies have evaluated maternal CRP levels in relation to neonatal complications [25,26,27]. Elevated CRP has been associated with PTB and LBW in multiple reports, suggesting that systemic inflammation may trigger early labor and restrict fetal growth [28,29,30]. However, associations between high CRP levels with SGA and stillbirth are less consistent [31]. Some studies report elevated risks, while others find no significant relationship [32]. These discrepancies may be explained by differences in study design, population characteristics, timing of CRP measurement, or laboratory methods. As a result, the overall strength and reliability of the evidence remain uncertain [33].

Given the burden of neonatal complications and the potential role of maternal inflammation in their development, a systematic review and meta-analysis is warranted. No prior synthesis has comprehensively evaluated maternal CRP levels across PTB, LBW, SGA, and stillbirth. Clarifying these associations could improve our understanding of inflammatory mechanisms in pregnancy, help refine risk stratification, and inform preventive strategies. This systematic review and meta-analysis therefore aim to critically appraise and pool the available evidence on maternal CRP as a biomarker of adverse neonatal outcomes.

## 2. Methods

### 2.1. Study Protocol

This meta-analysis adhered to rigorous methodological standards, incorporating both the MOOSE guidelines for observational studies and the PRISMA framework for systematic reviews [34,35,36]. The PRISMA checklist is found in Supplementary File S1. To minimize bias, two independent reviewers carried out the literature search, study selection, data extraction, and quality appraisal. Any discrepancies between reviewers were addressed through consultation with a third investigator. Additionally, the protocol for this review was prospectively registered with PROSPERO (CRD420251149463).

### 2.2. Search Strategy

This systematic review and meta-analysis study was conducted by reviewing articles from inception to July 2025. Articles were extracted using related keywords in most related to the field databases, including PubMed, Scopus, Web of Science, and Cochrane Library. We implemented the search strategy in English, according to the search engine, including the following keywords: “CRP” OR “hs-CRP” OR “C-reactive protein” OR “high-sensitivity C-reactive protein” OR “ultrasensitive CRP” OR “serum CRP” OR “plasma CRP” AND “Pregnancy Outcome” OR “Premature Birth” OR “Stillbirth” OR “Intrauterine Fetal Death” OR “IUFD” OR “Infant, Low Birth Weight” OR “Low Birth Weight” OR “LBW” OR “Fetal Growth Retardation” OR “IUGR” OR “Fetal Macrosomia” OR “Infant, Small for Gestational Age” OR “SGA” OR “Asphyxia Neonatorum” OR “Birth Asphyxia” (details on Supplementary File S2). This search was complemented by perusal of the references of related review studies and retrieved articles.

### 2.3. Eligibility Criteria and Study Selection

The included studies consisted of randomized and non-randomized prospective or retrospective cohorts and case–control investigations that evaluated the relationship between maternal CRP levels and neonatal outcomes. To be eligible, studies had to specifically investigate this correlation, while exclusion criteria encompassed unrelated or duplicate publications, studies where neonatal outcomes were not examined or were incorrectly defined, and those for which the full text was unavailable, in addition to review articles, case reports, case series, and editorials. Variables of interest in this study included PTB (delivery before 37 completed weeks of gestation) [37], LBW (birth weight less than 2500 g) [38], SGA (birth weight below the 10th percentile for gestational age) [39], and stillbirth (fetal death at or after 20 weeks of gestation) [40].

### 2.4. Data Extraction, Quality of Studies, and Certainty of Evidence

Two reviewers independently performed data extraction and quality assessment in duplicate, resolving disagreements through discussion and contacting study authors when clarification was needed. Extracted data included: (i) study characteristics such as setting, country, population demographics, and trimester of CRP measurement; (ii) outcome definitions and timing for PTB, LBW, SGA, and stillbirth; (iii) publication and exposure details including year, type of CRP measured (e.g., hs-CRP), and cut-off values for elevated CRP; (iv) analytical variables necessary for synthesis; and (v) special considerations such as prioritizing intended over actual mode of delivery when both were reported. Risk of bias was assessed separately using the Newcastle–Ottawa Scale (NOS) [41], which evaluates observational studies across three domains: (i) selection of study groups, (ii) comparability of groups, and (iii) ascertainment of exposure or outcome, with higher scores indicating lower risk of bias. In addition, the overall certainty of evidence for each outcome was graded using the GRADE approach [42], considering risk of bias, inconsistency, indirectness, imprecision, and publication bias.

### 2.5. Statistical Analysis

Meta-analyses were conducted using the DerSimonian and Laird random-effects model [43] in Stata 18.0 (Stata Statistical Software: Release 18. College Station, TX, USA: StataCorp LLC; 2023). A random-effects approach was selected due to expected heterogeneity in study designs and populations. For continuous variables such as maternal CRP levels, effect sizes were expressed as standardized mean differences (SMDs), while dichotomous outcomes were reported as odds ratios (ORs) with corresponding 95% confidence intervals (CIs). When studies reported outcomes across different time periods or distinct variables, these were pooled separately. Subgroup analyses were performed based on maternal age, CRP type, CRP cut-offs, and the trimester during which CRP was measured. Sensitivity analyses were conducted to examine the influence of individual studies on pooled results where possible. Statistical heterogeneity was assessed using the χ^2^ test for homogeneity and quantified with the I^2^ statistic, with I^2^ ≥ 50% considered indicative of substantial heterogeneity [44]. Publication bias was evaluated by visual inspection of funnel plots, supplemented by Begg’s rank correlation test and Egger’s regression test.

## 3. Result

### 3.1. Study Selection

**Figure 1:**
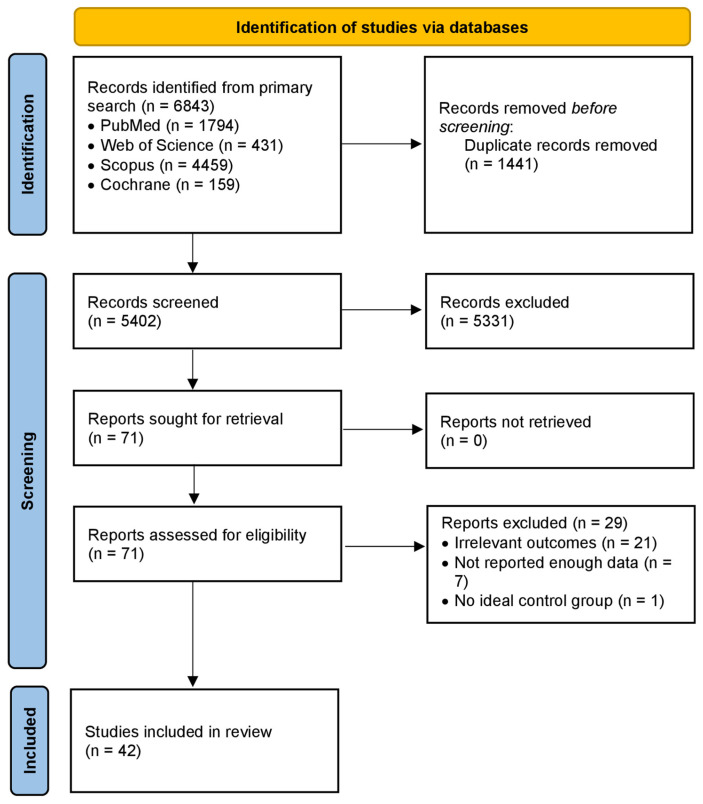
The PRISMA flowchart for literature search and study selection.

Our initial database search yielded 6843 records (PubMed = 1794, Web of Science = 431, Scopus = 4459, Cochrane = 159). After removing 1441 duplicates, a total of 5402 records were screened. Of these, 5331 records were excluded following title and abstract review. We assessed 71 full-text articles for eligibility, of which 29 were excluded (21 due to irrelevant outcomes, 7 for insufficient data reporting, and 1 for lack of an appropriate control group) [31,45,46,47,48,49,50,51,52,53,54,55,56,57,58,59,60,61,62,63,64,65,66,67,68,69,70,71,72]. Eventually, 42 studies [25,26,27,28,32,73,74,75,76,77,78,79,80,81,82,83,84,85,86,87,88,89,90,91,92,93,94,95,96,97,98,99,100,101,102,103,104,105,106,107,108,109] met the inclusion criteria and were included in the systematic review and meta-analysis. The study selection process of this review is presented in Figure 1.

### 3.2. Main Characteristics of the Included Studies

A total of 18,393 pregnant women were included across 42 studies conducted in Asia (India, China, South Korea, Indonesia, Iran, Iraq), Europe (UK, Spain, Netherlands, Denmark, Poland, Bulgaria, Ireland, Turkey), North America (USA), South America (Brazil), and Africa (Egypt). Sample sizes ranged from 48 participants in Indonesia to 6016 participants in the Netherlands. Most studies were prospective cohorts (*n* = 26), with additional designs including case–control (*n* = 12), retrospective cohort (*n* = 2), cross-sectional (*n* = 2), and nested case–control/nested cohort (*n* = 2). CRP was measured at different gestational stages: 1st trimester (*n* = 10 studies), 2nd trimester (*n* = 11), 3rd trimester (*n* = 13), and mixed trimesters (*n* = 8). Regarding biomarker type, 28 studies assessed CRP and 14 measured hs-CRP. Participant ages ranged from the youngest reported (18–20 years in India) to the oldest (median of 37.9 years in the UK), with most studies including women in their mid-to-late twenties or early thirties. All study characteristics are summarized in Table 1.

### 3.3. Risk of Bias (NOS Assessment)

The methodological quality of the 42 included studies, assessed using the NOS, ranged from 4/9 to 9/9 stars. A total of 14 studies achieved the maximum score of 9/9, indicating low risk of bias. 10 studies scored 8/9, 6 studies scored 7/9, 6 studies scored 6/9, 5 studies scored 5/9, and 1 study scored 4/9. In the selection domain, 42/42 studies received ≥2 stars, reflecting adequate case and control selection. In the comparability domain, only 15 studies earned the maximum of 2 stars, while the remaining 27 studies received 0–1 star, indicating limited adjustment for confounders. In the outcome domain, 38 studies received 3 stars, while 4 studies were rated with 2 stars. These findings show that although a substantial proportion of studies were of high methodological quality, inadequate control for potential confounders was the most frequent limitation (Table 1).

### 3.4. Quantitative Synthesis

**Figure 2:**
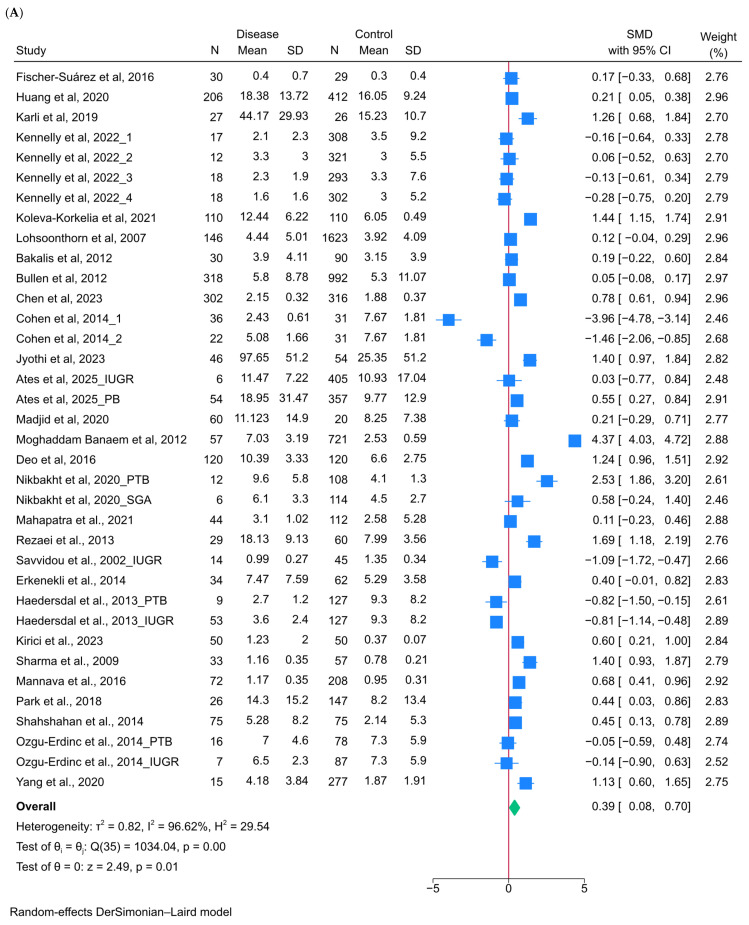

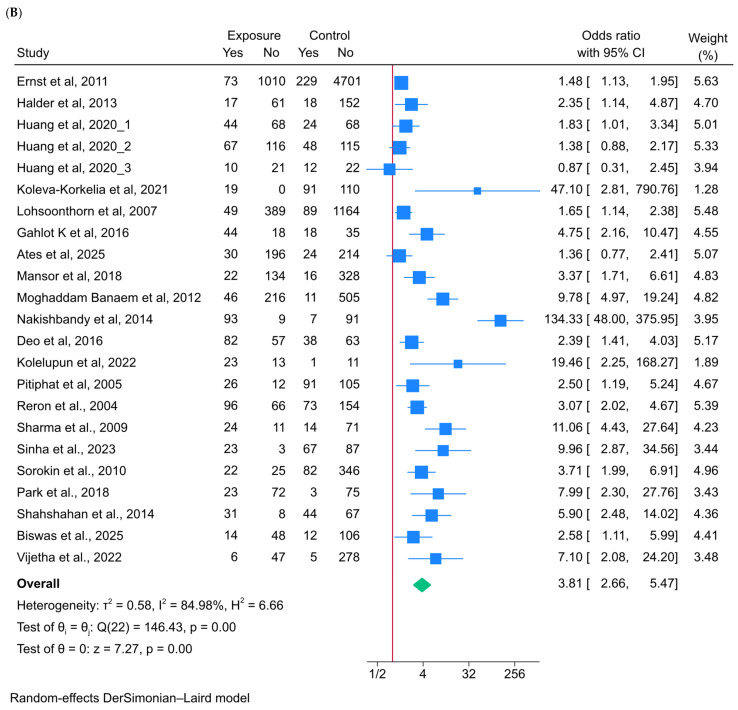

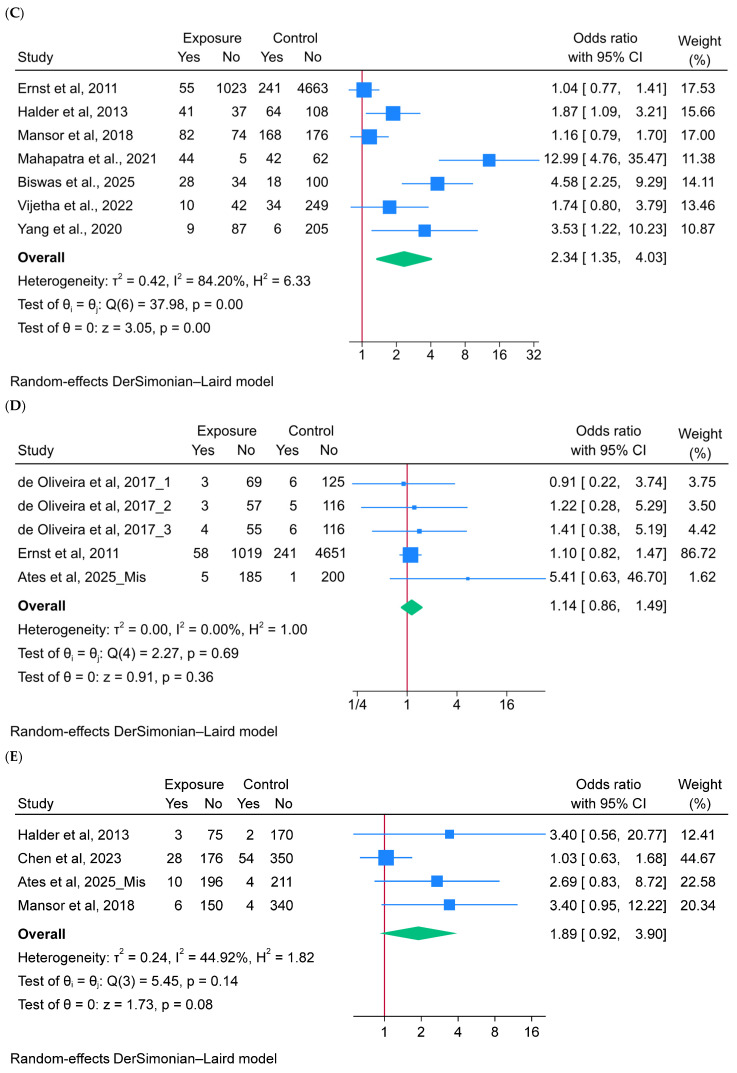
Forest plots showing the association between maternal C-reactive protein (CRP) levels and adverse neonatal outcomes [25,26,27,28,32,73,74,75,76,77,78,79,80,81,82,83,84,85,86,87,88,89,90,91,92,93,94,95,96,97,98,99,100,101,102,103,104,105,106,107,108,109]. (**A**) Standardized Mean Difference (SMD) of maternal CRP levels in overall adverse neonatal outcomes compared with controls. (**B**) Odds of Preterm Birth in relation to elevated maternal CRP. (**C**) Odds of Low Birth Weight (LBW) in relation to elevated maternal CRP. (**D**) Odds of Small for Gestational Age (SGA) in relation to maternal CRP. (**E**) Odds of Stillbirth in relation to maternal CRP.

The pooled analysis demonstrated that maternal CRP levels were significantly higher in pregnancies with adverse neonatal outcomes compared with controls (SMD, 0.39; 95% CI, 0.08–0.70; *p* = 0.01; I^2^ = 96.6%, *n* = 28) (Figure 2A). Subgroup analyses showed that this association was strongest in women aged 20–30 years (SMD = 0.74; 95% CI: 0.28–1.21) and in studies measuring hs-CRP (SMD = 0.87; 95% CI: 0.15–1.58), as well as when CRP was assessed in the third trimester (SMD = 0.69; 95% CI: 0.28–1.10) (Table 2). Elevated maternal CRP was strongly associated with increased odds of PTB (OR = 3.81; 95% CI: 2.66–5.47; *p* < 0.001; I^2^ = 85%; *n* = 23) (Figure 2B). Subgroup analyses revealed stronger associations in studies using hs-CRP (OR = 6.84; 95% CI: 2.69–17.44), in those with CRP cut-offs of 3.0–5.9 mg/L (OR = 6.51; 95% CI: 3.20–13.25), and when CRP was measured in the third trimester (OR = 6.14; 95% CI: 2.88–13.09) (Table 2). A significant association was also observed between elevated CRP and LBW (OR = 2.34; 95% CI: 1.35–4.03; *p* = 0.002; I^2^ = 84.2%; *n* = 7) (Figure 2C). Subgroup analyses indicated that significant associations were evident for studies using conventional CRP (OR, 2.39; 95% CI: 1.08–5.30) and for those with CRP cut-offs of 6–8.9 mg/L (OR: 1.95; 95% CI, 1.11–3.44), with the effect being most pronounced when CRP was measured in the second trimester (OR = 2.89; 95% CI: 1.06–7.94) (Table 2). By contrast, no significant association was identified for SGA (OR = 1.14; 95% CI: 0.86–1.49; *p* = 0.36; I^2^ = 0%; *n* = 5) (Figure 2D). Subgroup analyses stratified by maternal age did not demonstrate significant results, with both younger (OR = 1.68; 95% CI: 0.42–6.67) and older (OR = 1.17; 95% CI: 0.52–2.61) groups showing null associations (Table 2). Similarly, no significant association was found for stillbirth (OR = 1.89; 95% CI: 0.92–3.90; *p* = 0.08; I^2^ = 44.9%; *n* = 4) (Figure 2E).

Sensitivity analyses, including leave-one-out testing, did not materially alter the pooled estimates for any of the outcomes, and detailed results are presented in Supplementary File S3. Moreover, Evidence of publication bias was detected for overall CRP levels (Egger’s *p* = 0.022) and for PTB (Egger’s and Begg’s *p* < 0.001), with possible bias also suggested for LBW (Egger’s *p* < 0.001). No significant publication bias was observed for SGA (Egger’s *p* = 0.388; Begg’s *p* = 0.221) or stillbirth (Egger’s *p* = 0.024; Begg’s *p* = 0.734) (Supplementary File S4). The certainty of evidence, assessed using GRADE, was rated as moderate for overall neonatal complications, PTB, and LBW, primarily downgraded due to inconsistency (I^2^ > 60%). In contrast, the certainty was low for SGA and stillbirth, reflecting limitations from indirectness (few studies) and imprecision (wide CIs) (Supplementary File S5).

## 4. Discussion

However, there are systematic reviews that have evaluated the correlation between CRP levels and maternal complications [23,110,111]. To the best of our knowledge, this is the first systematic review and meta-analysis to comprehensively evaluate the correlation between maternal CRP levels and neonatal complications. Our systematic review and meta-analysis of 42 observational studies involving 18,393 pregnant women show that elevated maternal CRP is significantly associated with PTB and LBW, but not with SGA or stillbirth. These findings highlight the importance of systemic inflammation during pregnancy as a risk factor for adverse neonatal outcomes, suggesting that CRP may have a role both as a biomarker for risk stratification and as a target for future interventions in obstetric care.

Our findings are broadly in alignment with Nikbakht et al. [26] and Lohsoonthorn et al. [91], who both reported that higher maternal CRP levels were associated with increased risks of PTB and impaired fetal growth. Similarly, Zhang et al. observed significant associations between elevated maternal CRP and adverse offspring outcomes in the context of neuropsychiatric disorders, further supporting the role of maternal inflammation in developmental health [112]. Lucaroni et al. [113] further demonstrated, through an umbrella review of biomarkers for spontaneous PTB, that CRP measured in maternal plasma was associated with an odds ratio of 2.0 for PTB, while CRP measured in amniotic fluid was associated with an odds ratio of 8.0, findings that reinforce our pooled estimates of CRP as a marker of prematurity risk. Wei et al. [114] also showed in a systematic review of inflammatory cytokines that elevated CRP in amniotic fluid was strongly associated with spontaneous PTB (OR = 7.85; 95% CI: 3.88–15.87), whereas plasma CRP showed only a modest association (OR = 1.53; 95% CI: 1.22–1.90), highlighting the stronger predictive role of local inflammatory responses at the maternal–fetal interface compared to systemic inflammation. By contrast, van de Laar et al. [115] concluded that maternal CRP had only moderate predictive accuracy for histological chorioamnionitis and was not a reliable predictor of neonatal sepsis in women with PPROM, highlighting that its predictive value may vary depending on clinical context and outcomes assessed. In addition, some studies such as Brown et al. [116] and Chudal et al. [117] reported no significant associations between maternal CRP and adverse offspring outcomes, which may be explained by differences in study design, smaller sample sizes, timing of CRP measurement, and the specific outcomes investigated compared with our review.

Several biological pathways may explain the associations observed between elevated maternal CRP and adverse neonatal outcomes. CRP is an acute-phase reactant primarily induced by cytokines such as IL-6 and TNF-α, which amplify downstream inflammatory cascades [118,119]. In pregnancy, this systemic response can accelerate prostaglandin synthesis through COX-2 activation, promote cervical remodeling and uterine contractility, and ultimately preterm labor [120,121]. Park et al. demonstrated that maternal plasma IL-6 strongly correlated with serum CRP and amniotic fluid IL-6, and independently predicted intra-amniotic infection and imminent preterm delivery, underscoring the link between cytokine activation, systemic CRP, and the timing of labor [27]. Subclinical intrauterine infections may further account for elevated CRP in women without overt clinical symptoms [122]. Even low-grade infections can trigger maternal and fetal immune responses, compromise placental integrity, and promote premature rupture of membranes, all of which increase the risk of PTB [17,123]. Lohsoonthorn et al. observed that CRP elevations were particularly associated with very early and medically indicated PTB, suggesting that inflammation is especially relevant in the most severe forms of prematurity [91]. This interpretation is strengthened by Mendelian randomization findings from Chen et al., which showed that genetically elevated maternal CRP increased the risk of PTB and reduced birth weight, supporting a causal link rather than simple correlation [124].

In addition to its role in preterm delivery, inflammation associated with elevated CRP can impair placental perfusion and vascular remodeling, disrupt trophoblast invasion, and interfere with nutrient and oxygen transport [125,126]. These processes contribute to fetal growth restriction, manifesting clinically as LBW [127]. Ernst et al. reported that higher CRP levels early in pregnancy predicted growth restriction, supporting placental dysfunction as a central mechanism [32]. Evidence of abnormal uteroplacental blood flow in women with elevated CRP further reinforces the link between maternal inflammation, impaired placental function, and restricted fetal growth [128]. Taken together, these data suggest that CRP reflects a systemic “read-out” of inflammatory pathways operating at both the maternal–fetal interface and in maternal circulation. Elevated CRP may therefore capture processes ranging from infection-related membrane weakening to placental insufficiency and cytokine-driven activation of labor. The stronger associations observed with hs-CRP assays and later gestational sampling in our subgroup analyses are consistent with the heightened role of low-grade inflammation and localized immune activation as pregnancy progresses.

Our review has several strengths. We included a large number of studies (*n* = 42), with an overall large, combined sample size, and incorporated both standard CRP and hs-CRP data, with stratified analyses by trimester, age, and CRP cut-offs. We also assessed risk of bias using NOS and certainty via GRADE and conducted sensitivity analyses. However, several limitations must also be noted. Included studies are observational and thus may be subject to residual confounding; many did not adjust fully for all potential confounders, as our NOS assessment showed. There is also very high heterogeneity in many pooled estimates (e.g., I^2^ over 80% in PTB and LBW), which may reflect differences in study design, CRP measurement, sample frames, and population characteristics. Publication bias was evident for some outcomes, meaning our effect estimates might be inflated. Another limitation is that all included studies measured maternal serum or plasma CRP, whereas evidence suggests that local inflammatory markers in amniotic fluid may provide stronger predictive value for adverse outcomes, highlighting that our review may underestimate the role of intrauterine inflammation. As this meta-analysis was limited to outcomes most consistently reported in the literature, fewer studies evaluated fetal growth restriction using standardized definitions; therefore, LBW and SGA were analyzed as growth-related outcomes, which may partially overlap with PTB and may not fully represent pathological fetal growth restriction. Finally, data on stillbirth and SGA remain sparse, limiting precision and certainty of evidence for those outcomes.

The findings of this systematic review and meta-analysis have important implications for clinical practice, policy, and future research. Clinically, routine measurement of maternal CRP—particularly using high-sensitivity assays—may aid in identifying pregnant women at increased risk of preterm birth and low birth weight, allowing for closer monitoring, early interventions, and potential use of anti-inflammatory or infection-targeted therapies. From a policy perspective, these results support integrating inflammatory biomarker screening into prenatal care guidelines, especially in high-risk populations, while emphasizing the need for standardized CRP thresholds and timing of assessment. For future research, large, well-controlled prospective studies and Mendelian randomization analyses are needed to clarify causal mechanisms, refine CRP cut-offs for clinical use, and explore whether modulation of maternal inflammation can improve neonatal outcomes. Additionally, investigation into local inflammatory markers in amniotic fluid may provide deeper insights into intrauterine processes driving adverse pregnancy outcomes.

## 5. Conclusions

In conclusion, this systematic review and meta-analysis provides evidence that elevated maternal CRP is associated with increased risks of PTB and LBW but does not appear reliably associated with SGA or stillbirth. These results support the potential of CRP as a maternal biomarker for identifying pregnancies at risk, especially when measured with hs-CRP in the later trimesters. Future research should prioritize well-designed prospective cohorts, harmonization of CRP measurement and cut-off points, and exploring interventions to reduce inflammation during pregnancy in order to improve neonatal outcomes.

## Figures and Tables

**Table 1:** Main Characteristics of included studies.

Study	Country	Sample Size (*N*)	Study Design	Trimester of CRP Evaluation	CRP Type	Age of Participants (years)		Neonatal Complications Evaluated	Newcastle–Ottawa Scale (NOS) Assessment			
						Cases	Controls		Selection	Comparability	Outcome	Total
Ates et al., 2025 [25]	Turkey	411	Prospective cohort	Mix	CRP	30.7 ± 5.4	29.1 ± 4.9	PTB, IUGR	★★★	★★☆	★★★	8/9
Bakalis et al., 2012 [73]	UK	120	Prospective cohort	1st	hs-CRP	33.0 (29.2–37.9) (median, IQR)	32.9 (28.0–36.7) (median, IQR)	PTB	★★★	★★☆	★★★	8/9
Biswas et al., 2025 [74]	India	180	Prospective cohort	3rd	hs-CRP	21.9 ± 4.3	21.9 ± 3.3	LBW, PTB, IUGR	★★★	★★★	★★★	9/9
Bullen et al., 2012 [75]	USA	1310	Prospective cohort	2nd	CRP	NR	NR	PTB	★★★	★★★	★★★	9/9
Chen et al., 2023 [76]	China	618	Prospective cohort	1st	hs-CRP	33.7	32.6	PTB	★★★	★★★	★★★	9/9
Cohen et al., 2014 [77]	Israel	89	Prospective observational	1st	CRP	29.0 ± 7.6	32.2 ± 4.8	IUGR	★★★	★☆☆	★★★	7/9
de Oliveira et al., 2017 [78]	Brazil	203	Prospective Cohort	Mix	hs-CRP	20–40		SGA	★★★	★★☆	★★★	8/9
Deo et al., 2016 [79]	India	240	Case–Control	Mix	CRP	25.3 ± 3.1	24.9 ± 3.3	PTB	★★☆	★☆☆	★★☆	5/9
Erkenekli et al., 2014 [80]	Turkey	96	Case–Control	3rd	CRP	26.3 ± 5.1	26.82 ± 5.27	IUGR	★★★	★★☆	★★★	8/9
Ernst et al., 2011 [32]	Netherlands	6016	Prospective Cohort	1st	hs-CRP	29.8 ± 5.1		PTB, LBW, SGA	★★★	★★★	★★★	9/9
Fischer-Suárez et al., 2016 [81]	Spain	123	Prospective Case–Control	3rd	hs-CRP	27.2 ± 6.9	31.8 ± 4.0	PTB	★★★	★☆☆	★★☆	6/9
Gahlot et al., 2016 [82]	India	112	Prospective Cohort	Mix	CRP	21–25		PTB	★★☆	★☆☆	★★☆	5/9
Haedersdal et al., 2013 [83]	Denmark	218	Case–Control	2nd	CRP	26.9 (18.2–37.3) (Median, range)	27.5 (17.5–37.8) (Median, range)	PTB, IUGR	★★★	★★☆	★★★	8/9
Halder et al., 2013 [84]	India	250	Prospective Cohort	1st	CRP	NR	NR	PTB, LBW, IUGR	★★★	★☆☆	★★☆	6/9
Huang et al., 2020 [28]	China	618	Case–Control	Mix	CRP	28.3 ± 5.3	28.2 ± 4.7	PTB	★★★	★★★	★★★	9/9
Jyothi et al., 2023 [85]	India	100	Prospective Cohort	2nd	CRP	25.4 ± 4.0	26.4 ± 3.4	PTB, LBW	★★★	★★☆	★★★	8/9
Karli et al., 2019 [86]	Turkey	53	Prospective Case–Control	3rd	CRP	31 (27–34) (Median, IQR)	28.5 (24.7–32) (Median, IQR)	IUGR, LBW	★★☆	★★☆	★★★	7/9
Kennelly et al., 2022 [87]	Ireland	406	Prospective Cohort	2nd	CRP	32.9 ± 4.6	32.2 ± 4.1	PTB, LBW, SGA	★★★	★★★	★★★	9/9
Kirici et al., 2023 [88]	Turkey	100	Case–Control	3rd	hs-CRP	28 (25–31) (Median, IQR)	29 (25–32) (Median, IQR)	IUGR, LBW	★★☆	★★☆	★★★	7/9
Kolelupun et al., 2022 [89]	Indonesia	48	Case–Control	3rd	hs-CRP	27 (17–34) (Median, range)	28 (17–34) (Median, range)	PTB	★★☆	★☆☆	★★☆	5/9
Koleva-Korkelia et al., 2021 [90]	Bulgaria	220	Case–Control	3rd	CRP	31.8 ± 4.1	26.9 ± 5.8	PTB	★★★	★☆☆	★★☆	6/9
Lohsoonthorn et al., 2007 [91]	USA	1769	Prospective Cohort	1st	CRP	32.2 ± 0.5	32.1 ± 0.1	PTB	★★★	★★★	★★★	9/9
Madjid et al., 2020 [92]	Indonesia	80	Case–Control	3rd	CRP	NR	NR	PTB	★★☆	★☆☆	★★☆	5/9
Mahapatra et al., 2021 [93]	India	156	Prospective Cohort	Mix	CRP	26.6 ± 3.9		PTB, LBW	★★★	★★★	★★★	9/9
Mannava et al., 2016 [94]	India	210	Prospective Cohort	2nd	CRP	20–35		PTB	★★☆	★☆☆	★★☆	5/9
Mansor & Farag, 2018 [95]	Egypt	500	Prospective Cohort	2nd	hs-CRP	NR	NR	PTB, LBW	★★★	★☆☆	★★☆	6/9
Moghaddam Banaem et al., 2012 [96]	Iran	778	Prospective Cohort	1st	hs-CRP	26 (23–28) (Median, 95% CI)	26 (25–26) (Median, 95% CI)	PTB	★★★	★★★	★★★	9/9
Nakishbandy & Barawi, 2014 [97]	Iraq	200	Case–Control	2nd	hs-CRP	27.7 ± 5.9	28.9 ± 6.1	PTB	★★☆	★☆☆	★★☆	5/9
Nikbakht et al., 2020 [26]	Iran	120	Prospective Cohort	1st	CRP	26.5 ± 4.4		PTB, SGA	★★★	★★☆	★★★	8/9
Ozgu-Erdinc et al., 2014 [98]	Turkey	94	Prospective Cohort	2nd	hs-CRP	35 (23–40) (Median, range)	35 (19–44) (Median, range)	PTB, IUGR	★★★	★☆☆	★★☆	6/9
Park et al., 2018 [27]	South Korea	173	Retrospective Cohort	3rd	CRP	33.0 ± 4.0	31.3 ± 4.0	PTB	★★★	★★☆	★★★	8/9
Pitiphat et al., 2005 [99]	USA	234	Nested Case–Control	1st	hs-CRP	NR	NR	PTB	★★★	★★★	★★★	9/9
Reron et al., 2004 [100]	Poland	389	Retrospective Cohort	3rd	CRP	27.5 ± 6.1	27.5 ± 6.1	PTB	★★☆	★☆☆	★★☆	5/9
Rezaei et al., 2013 [101]	Iran	89	Nested Cohort	3rd	CRP	22.3 ± 3.7	22.7 ± 2.6	PTB	★★★	★★☆	★★★	8/9
Rzepka et al., 2016 [102]	Poland	211	Prospective Cohort	3rd	CRP	30.2 ± 6.2	27.9 ± 5.9	PTB	★★★	★★★	★★★	9/9
Savvidou et al., 2002 [103]	UK	90	Cross-sectional	2nd	hs-CRP	26.9 ± 6.0	29 ± 5.5	IUGR	★★★	★★☆	★★★	8/9
Shahshahan et al., 2014 [104]	Iran	150	Prospective Cohort	3rd	CRP	28.1 ± 4.6	27.1 ± 4.9	PTB	★★★	★☆☆	★★★	7/9
Sharma et al., 2009 [105]	India	90	Prospective Cohort	2nd	CRP	18–35		PTB	★★☆	★☆☆	★★☆	5/9
Sinha et al., 2023 [106]	India	180	Cross-sectional	3rd	CRP	26.5 ± 4.1	26.1 ± 3.1	PTB	★★☆	★☆☆	★★☆	5/9
Sorokin et al., 2010 [107]	USA	475	Prospective Cohort	3rd	CRP	NR	NR	PTB	★★★	★★☆	★★★	8/9
Vijetha et al., 2022 [108]	India	359	Prospective Cohort	2nd	CRP	20–30		PTB, LBW, IUGR, Stillbirth	★★☆	★☆☆	★☆☆	4/9
ang et al., 2020 [109]	China	307	Prospective Cohort	2nd	hs-CRP	28.3 ± 3.9	28.3 ± 3.1	LBW	★★★	★★☆	★★★	8/9

PTB: Preterm Birth, LBW: Low Birth Weight, IUGR: Intrauterine Growth Restriction, SGA: Small for Gestational Age, CRP: C-Reactive Protein, hs-CRP: High-Sensitivity C-Reactive Protein, NR: Not Reported.

**Table 2:** Subgroup analysis of included studies.

Variable	Sub-Grouped by		No. of Studies	Effect Size (SMD)	95% CI	I^2^ (%)	*p* for Heterogeneity
CRP levels and neonatal complications	Age	Young adult (20–30)	20	0.74	0.28, 1.21	97.40	0.000
		Middle-aged adult (31–50)	15	−0.08	−0.47, 0.31	94.40	0.000
	Neonatal Complication	Preterm Birth (PTB)	21	0.77	0.40, 1.14	97.22	0.000
		IUGR	8	−0.44	−1.30, 0.42	96.32	0.000
		SGA	3	−0.05	−0.46, 0.36	37.71	0.200
		LBW	2	0.60	−0.39, 1.59	89.89	0.000
	CRP type	CRP	23	0.14	−0.15, 0.43	94.40	0.000
		hs-CRP	13	0.87	0.15, 1.58	97.58	0.000
	Pregnancy Trimester	1st	10	0.31	−0.48, 1.09	98.6	0.000
		2nd	13	0.23	−0.12, 0.59	91.0	0.000
		3rd	9	0.69	0.28, 1.10	89.2	0.000
		Mix	2	0.43	0.00, 0.87	30.8	0.230
**Variable**	**Sub-Grouped by**		**No. of Studies**	**Effect Size (OR)**	**95% CI**	**I^2^ (%)**	***p* for** **Heterogeneity**
Preterm birth (PTB)	Age	Young adult (20–30)	18	3.94	2.55, 6.07	87.2	0.000
		Middle-aged adult (31–50)	5	3.24	1.69, 6.22	68.4	0.010
	CRP Type	CRP	15	2.72	1.98, 3.73	67.3	0.000
		hs-CRP	8	6.84	2.69, 17.44	93.1	0.000
	CRP cut-off	3.0–5.9 mg/L	2	6.51	3.20, 13.25	0.0	0.700
		6–8.9 mg/L	15	4.71	2.89, 7.66	86.1	0.000
		≥9 mg/L	6	1.83	1.19, 2.81	66.0	0.010
	Pregnancy Trimester	1st	6	2.41	1.49, 3.89	81.6	0.000
		2nd	6	4.26	2.15, 8.45	79.1	0.000
		3rd	11	6.14	2.88, 13.09	86.7	0.000
Small for gestational age (SGA)	Age	Young adult (20–30)	2	1.68	0.42, 6.67	51.4	0.150
		Middle-aged adult (31–50)	3	1.17	0.52, 2.61	0.0	0.900
Low-birth weight (LBW)	CRP Type	CRP	4	2.39	1.08, 5.30	84.8	0.000
		hs-CRP	3	2.43	0.80, 7.40	88.5	0.000
	CRP cut-off	3.0–5.9 mg/L	2	6.86	1.92, 24.55	67.1	0.080
		6–8.9 mg/L	4	1.95	1.11, 3.44	73.9	0.010
	Pregnancy Trimester	1st trimester	2	1.33	0.76, 2.35	71.0	0.060
		2nd trimester	4	2.89	1.06, 7.94	86.0	0.000

## Data Availability

All data are available within the manuscript and its Supplementary Files.

## Supplementary Materials

The following supporting information can be downloaded at: https://www.mdpi.com/article/10.3390/jcm15062114/s1. Supplementary File S1: The PRISMA checklist; Supplementary File S2: The search strategy [31,45,46,47,48,49,50,51,52,53,54,55,56,57,58,59,60,61,62,63,64,65,66,67,68,69,70,71,72].; Supplementary File S3: Sensitivity analysis of the association between maternal c-reactive protein (CRP) levels and adverse neonatal outcomes: (A) standardized mean difference (SMD) of maternal CRP levels in overall adverse neonatal outcomes compared with controls, (B) odds of preterm birth in relation to elevated maternal CRP, (C) odds of low birth weight in relation to elevated maternal CRP, (D) odds of small for gestational age in relation to maternal CRP, and (E) odds of stillbirth in relation to maternal CRP; Supplementary File S4: Funnel plots assessing publication bias for the association between maternal CRP levels and adverse neonatal outcomes, with Egger’s and Begg’s test *p*-values reported for each outcome: (A) standardized mean difference (SMD) of maternal CRP levels in overall adverse neonatal outcomes compared with controls, Egger’s *p* = 0.022, Begg’s *p* = 0.334, (B) odds of preterm birth in relation to elevated maternal CRP, Egger’s *p* < 0.001, Begg’s *p* < 0.001, (C) odds of low birth weight in relation to elevated maternal CRP, Egger’s *p* < 0.001, Begg’s *p* = 0.072, (D) odds of small for gestational age in relation to maternal CRP, Egger’s *p* = 0.388, Begg’s *p* = 0.221, and (E) odds of stillbirth in relation to maternal CRP; Supplementary File S5: Certainty of evidence based on GRADE approach for the analysis of the association between maternal CRP levels and adverse neonatal outcomes.
