# Supplementary material for: Association Between Maternal C-Reactive Protein (CRP) Levels and Adverse Neonatal Outcomes: A Systematic Review and Meta-Analysis
Source: J Clin Med. 2026 Mar 10;15(6):2114. doi: 10.3390/jcm15062114 (PMC13027311; doi:10.3390/jcm15062114)
Supplement: Supplementary file 1 [file jcm-15-02114-s001.zip › CRP_Supplementary File S2.pdf]

Supplementary File S2. The search strategy.

Maternal serum homocysteine levels correlation with infant and pregnancy outcomes: A systematic review and dose–response meta-analysis

Groups	Descriptors
Outcome	“Pregnancy Outcome” OR “Premature Birth” OR “Stillbirth” OR “Intrauterine Fetal Death” OR “IUFD” OR “Infant, Low Birth Weight” OR “Low Birth Weight” OR “LBW” OR “Fetal Growth Retardation” OR “IUGR” OR “Fetal Macrosomia” OR “Infant, Small for Gestational Age” OR “SGA” OR “Asphyxia Neonatorum” OR “Birth Asphyxia”
Exposure	"CRP" OR "hs-CRP" OR "C-reactive protein" OR "high-sensitivity C-reactive protein" OR "ultrasensitive CRP" OR "serum CRP" OR "plasma CRP"

PUBMED
Number of localized studies: 1794
Limits: -
Number of studies after applying limits: 1794

	Descriptors	Number of studies reached
**#1**	"pregnancy outcome"[MeSH Terms] OR ("pregnancy"[All Fields] AND "outcome"[All Fields]) OR "pregnancy outcome"[All Fields] OR ("premature birth"[MeSH Terms] OR ("premature"[All Fields] AND "birth"[All Fields]) OR "premature birth"[All Fields]) OR ("stillbirth"[MeSH Terms] OR "stillbirth"[All Fields] OR "stillbirths"[All Fields]) OR ("stillbirth"[MeSH Terms] OR "stillbirth"[All Fields] OR ("intrauterine"[All Fields] AND "fetal"[All Fields] AND "death"[All Fields]) OR "intrauterine fetal death"[All Fields] OR "fetal death"[MeSH Terms] OR ("fetal"[All Fields] AND "death"[All Fields]) OR "fetal death"[All Fields]) OR "IUFD"[All Fields] OR ("infant, low birth weight"[MeSH Terms] OR ("infant"[All Fields] AND "low"[All Fields] AND "birth"[All Fields] AND "weight"[All Fields]) OR "low birth weight infant"[All Fields] OR "infant low birth weight"[All Fields]) OR ("infant, low birth weight"[MeSH Terms] OR ("infant"[All Fields] AND "low"[All Fields] AND "birth"[All Fields] AND "weight"[All Fields]) OR "low birth weight infant"[All Fields] OR ("low"[All Fields] AND "birth"[All Fields] AND "weight"[All Fields]) OR "low birth weight"[All Fields]) OR "LBW"[All Fields] OR ("foetal growth retardation"[All Fields] OR "fetal growth retardation"[MeSH Terms] OR ("fetal"[All Fields] AND "growth"[All Fields] AND "retardation"[All Fields]) OR "fetal growth retardation"[All Fields]) OR ("fetal growth retardation"[MeSH Terms] OR ("fetal"[All Fields] AND "growth"[All Fields] AND "retardation"[All Fields]) OR "fetal growth retardation"[All Fields] OR "iugr"[All Fields]) OR ("foetal macrosomia"[All Fields] OR "fetal macrosomia"[MeSH Terms] OR ("fetal"[All Fields] AND "macrosomia"[All Fields]) OR "fetal macrosomia"[All Fields]) OR ("infant, small for gestational age"[MeSH Terms] OR ("infant"[All Fields] AND "small"[All Fields] AND "gestational"[All Fields] AND "age"[All Fields]) OR "small for gestational age infant"[All Fields] OR "infant small for gestational age"[All Fields]) OR "SGA"[All Fields] OR ("asphyxia neonatorum"[MeSH Terms] OR ("asphyxia"[All Fields] AND "neonatorum"[All Fields]) OR "asphyxia neonatorum"[All Fields]) OR ("asphyxia neonatorum"[MeSH Terms] OR ("asphyxia"[All Fields] AND	342341

	"neonatorum"[All Fields]) OR "asphyxia neonatorum"[All Fields] OR ("birth"[All Fields] AND "asphyxia"[All Fields]) OR "birth asphyxia"[All Fields])	
**#2**	"C-Reactive Protein"[MeSH Terms] OR "C-Reactive Protein"[All Fields] OR "CRP"[All Fields] OR "hs-CRP"[All Fields] OR "high-sensitivity C-reactive protein"[All Fields] OR "ultrasensitive CRP"[All Fields] OR "serum CRP"[All Fields] OR "plasma CRP"[All Fields]	138153
**#3**	**#1** AND **#2**	1794

WEB OF SCIENCE
Number of localized studies: 473
Limits: documents types (articles)
Number of studies after applying limits: 431

	**Descriptors**	Number of studies reached
**#1**	TS=(“ Pregnancy Outcome”) OR TS=(“ Premature Birth”) OR TS=(“ Stillbirth”) OR TS=(“ Intrauterine Fetal Death”) OR TS=(“ IUFD”) OR TS=(“ Infant, Low Birth Weight”) AND TS=(“ Low Birth Weight”) OR TS=(“ LBW”) OR TS=(“ Fetal Growth Retardation”) OR TS=(“ IUGR”) OR TS=(“ Fetal Macrosomia”) AND TS=(“ Infant, Small for Gestational Age”) OR TS=(“ SGA”) OR TS=(“ Asphyxia Neonatorum”) OR TS=(“ Birth Asphyxia”)	55895
**#2**	TS=("CRP") OR TS=("hs-CRP") OR TS=("C-reactive protein") OR TS=("high-sensitivity C-reactive protein") OR TS=("ultrasensitive CRP") OR TS=("serum CRP") OR TS=("plasma CRP")	152855
**#3**	**#1** AND **#2**	473

SCOPUS
Number of localized studies: 5226
Limits: *document type* (article and article in press)
Number of studies after applying limits: 4459

	**Descriptors**	Number of studies reached
**#1**	(TITLE-ABS-KEY (birth AND asphyxia)) OR (TITLE-ABS-KEY (asphyxia AND neonatorum)) OR (TITLE-ABS-KEY (sga)) OR (TITLE-ABS-KEY (infant, AND small AND for AND gestational AND age)) OR (TITLE-ABS-KEY (fetal AND macrosomia)) OR (TITLE-ABS-KEY (pregnancy AND outcome)) OR (TITLE-ABS-KEY (premature AND birth)) OR (TITLE-ABS-KEY (stillbirth)) OR (TITLE-ABS-KEY (intrauterine AND fetal AND death)) OR (TITLE-ABS-KEY (iufd)) OR (TITLE-ABS-KEY (infant, AND low AND birth AND weight)) OR (TITLE-ABS-KEY (low AND birth AND weight)) OR (TITLE-ABS-KEY (lbw)) OR (TITLE-ABS-KEY (fetal AND growth AND retardation)) OR (TITLE-ABS-KEY (iugr))	456429
**#2**	(TITLE-ABS-KEY ("CRP")) OR (TITLE-ABS-KEY ("hs-CRP")) OR (TITLE-ABS-KEY ("C-reactive protein")) OR (TITLE-ABS-KEY ("high-	294499

	sensitivity C-reactive protein")) OR (TITLE-ABS-KEY ("ultrasensitive CRP")) OR (TITLE-ABS-KEY ("serum CRP")) OR (TITLE-ABS-KEY ("plasma CRP"))	
**#3**	**#1** AND **#2**	5226

COCHRANE
Number of localized studies: 159
Limits: -
Number of studies after applying limits: 159

	**Descriptors**	Number of studies reached
**#1**	Me ("Pregnancy Outcome"):ti,ab,kw or ("Premature Birth"):ti,ab,kw or ("Stillbirth"):ti,ab,kw or ("Intrauterine Fetal Death"):ti,ab,kw or ("IUFD"):ti,ab,kw or ("Infant, Low Birth Weight"):ti,ab,kw or ("Low Birth Weight"):ti,ab,kw or ("LBW"):ti,ab,kw or ("Fetal Growth Retardation"):ti,ab,kw or ("IUGR"):ti,ab,kw or ("Fetal Macrosomia"):ti,ab,kw or ("Infant, Small for Gestational Age"):ti,ab,kw or ("SGA"):ti,ab,kw or ("Asphyxia Neonatorum"):ti,ab,kw or ("Birth Asphyxia"):ti,ab,kw	13133
**#2**	Me ("CRP"):ti,ab,kw<br>OR ("hs-CRP"):ti,ab,kw<br>OR ("C-reactive protein"):ti,ab,kw<br>OR ("high-sensitivity C-reactive protein"):ti,ab,kw<br>OR ("ultrasensitive CRP"):ti,ab,kw<br>OR ("serum CRP"):ti,ab,kw<br>OR ("plasma CRP"):ti,ab,kw	27977
**#3**	**#1** AND **#2**	159

Excluded studies

	**Study**	**Reason**
1	Witteveen et al., 2022 [31]	No related variables
2	Antoniou et al, 2024 [45]	No related variables
3	Bucak et al, 2024 [46]	Not reported enough data
4	Chen et al, 2024 [47]	Not reported enough data
5	Christensen et al, 2023 [48]	No related variables
6	Gogeneni et al, 2015 [49]	No related variables
7	Kelly et al, 2022 [50]	No related variables
8	Khairnar et al, 2015 [51]	No related variables
9	Chen et al, 2022 [52]	Not reported enough data
10	Hackney et al, 2008 [53]	Not reported enough data
11	Hastie et al, 2011 [54]	No related variables
12	James et al, 2020 [55]	No related variables
13	Kidd et al, 2022 [56]	No related variables
14	Kim et al, 2011 [57]	No related variables
15	Kyaw et al, 2025 [58]	No related variables
16	Manoppo et al, 2017 [59]	No related variables

17	Ng et al, 1997 [60]	No related variables
18	Parvatikar et al, 2013 [61]	Not reported enough data
19	Paul et al, 2008 [62]	No related variables
20	Pieczyńska et al, 2020 [63]	No related variables
21	Keenan-Devlin et al, 2021 [64]	Not reported enough data
22	Ryu et al., 2017 [65]	No related variables
23	Aktar et al., 2024 [66]	No related variables
24	Ali et al, 2012 [67]	No related variables
25	Seyhanli et al, 2024 [68]	Not reported enough data
26	Shafiq et al, 2021 [69]	No related variables
27	Song et al, 2023 [70]	No related variables
28	Suwardewa et al., 2022 [71]	No Ideal Control group
29	Yeates et al., 2020 [72]	No related variables

REFERENCES as in the MAIN TEXT

31. Witteveen, A.B.; Henrichs, J.; Bellers, M.; van Oenen, E.; Verhoeven, C.J.; Vrijkotte, T.G. Mediating role of C-reactive protein in associations between pre-pregnancy BMI and adverse maternal and neonatal outcomes: the ABCD-study cohort. *The Journal of Maternal-Fetal & Neonatal Medicine* **2022**, *35*, 2867-2875.
45. Antoniou, M.-C.; Quansah, D.Y.; Gilbert, L.; Arhab, A.; Schenk, S.; Lacroix, A.; Stuijfzand, B.; Horsch, A.; Puder, J.J. Association between maternal and fetal inflammatory biomarkers and offspring weight and BMI during the first year of life in pregnancies with GDM: MySweetheart study. *Frontiers in endocrinology* **2024**, *15*, 1333755.
46. Bucak, M.; Seyhanli, Z.; Cakir, B.T.; Ulusoy, C.O.; Karabay, G.; Aktemur, G.; Akkus, F.; Yılmaz, Z.V. The role of the neutrophil-to-lymphocyte ratio (NLR), platelet-to-lymphocyte ratio (PLR), C-reactive protein (CRP) and fibrinogen in predicting the latent period after preterm premature rupture of membranes between 24 and 34 weeks. *Perinatal Journal* **2024**, *32*, 216-225.
47. Chen, Y.-C.S.; Mirzakhani, H.; Knihtilä, H.; Fichorova, R.N.; Luu, N.; Laranjo, N.; Jha, A.; Kelly, R.S.; Weiss, S.T.; Litonjua, A.A. The association of prenatal C-reactive protein and interleukin-8 levels with maternal characteristics and preterm birth. *American journal of perinatology* **2024**, *41*, e843-e852.
48. Christensen, S.H.; Rom, A.L.; Greve, T.; Lewis, J.I.; Frøkiær, H.; Allen, L.H.; Mølgaard, C.; Renault, K.M.; Michaelsen, K.F. Maternal inflammatory, lipid and metabolic markers and associations with birth and breastfeeding outcomes. *Frontiers in nutrition* **2023**, *10*, 1223753.
49. Gogeneni, H.; Buduneli, N.; Ceyhan‐Öztürk, B.; Gümüş, P.; Akcali, A.; Zeller, I.; Renaud, D.E.; Scott, D.A.; Özçaka, Ö. Increased infection with key periodontal pathogens during gestational diabetes mellitus. *Journal of clinical periodontology* **2015**, *42*, 506-512.
50. Kelly, R.S.; Lee-Sarwar, K.; Chen, Y.-C.; Laranjo, N.; Fichorova, R.; Chu, S.H.; Prince, N.; Lasky-Su, J.; Weiss, S.T.; Litonjua, A.A. Maternal inflammatory biomarkers during pregnancy and early life neurodevelopment in offspring: results from the VDAART study. *International Journal of Molecular Sciences* **2022**, *23*, 15249.
51. Khairnar, M.S.; Pawar, B.R.; Marawar, P.P.; Khairnar, D.M. Estimation of changes in C-reactive protein level and pregnancy outcome after nonsurgical supportive periodontal therapy in women affected with periodontitis in a rural set up of India. *Contemporary clinical dentistry* **2015**, *6*, S5-S11.
52. Chen, J.; Navais, P.S.; Xu, H.; Flatley, C.; Bacelis, J.; Monangi, N.; Kacerovsky, M.; Hallman, M.; Teramo, K.; Lawlor, D.A. Interrogating the causal effects of maternal circulating CRP on gestational duration and birth weight. *medRxiv* **2022**, 2022.2005. 2016.22275164.

53. Hackney, D.N.; Macpherson, T.A.; Dunigan, J.T.; Simhan, H.N. First-trimester maternal plasma concentrations of C-reactive protein in low-risk patients and the subsequent development of chorioamnionitis. *American journal of perinatology* **2008**, *25*, 407-411.
54. Hastie, C.E.; Smith, G.C.; Mackay, D.F.; Pell, J.P. Association between preterm delivery and subsequent C-reactive protein: a retrospective cohort study. *American journal of obstetrics and gynecology* **2011**, *205*, 556. e551-556. e554.
55. James, U.A.; Imaralu, J.O.; Esiaba, I. Evaluation of Serum Interleukin-6 and C-Reactive Protein Levels among Women During Term Labour.
56. Kidd, M.G.; McDade, T.W. Association between C‐reactive protein response to influenza vaccine during pregnancy and birth outcomes. *American Journal of Human Biology* **2022**, *34*, e23569.
57. Kim, H.; Hwang, J.; Ha, E.; Park, H.; Ha, M.; Lee, S.; Hong, Y.; Chang, N. Association of maternal folate nutrition and serum C-reactive protein concentrations with gestational age at delivery. *European journal of clinical nutrition* **2011**, *65*, 350-356.
58. Kyaw, E.M.M.; San, C. Association Between Maternal Serum C-Reactive Protein in Early Pregnancy and Spontaneous Preterm Delivery: A Prospective Hospital-Based Study in Yangon, Myanmar. **2025**.
59. Manoppo, M.; Tendean, H.M.; Sondakh, J.M. High Sensitivity CReactive Protein (hsCRP) Level on Premature Rupture of Membrane (PROM) at Term Pregnancy. *Indonesian Journal of Obstetrics and Gynecology* **2017**, 12-15.
60. Ng, P.; Cheng, S.; Chui, K.; Fok, T.; Wong, M.; Wong, W.; Wong, R.; Cheung, K. Diagnosis of late onset neonatal sepsis with cytokines, adhesion molecule, and C-reactive protein in preterm very low birthweight infants. *Archives of Disease in Childhood-Fetal and Neonatal Edition* **1997**, *77*, F221-F227.
61. Parvatikar, S. A Comparative Study of Serum Uric Acid C Reactive Protein and Serum Calcium in Preeclampsia and Normal Pregnancy. Rajiv Gandhi University of Health Sciences (India), 2013.
62. Paul, K.; Boutain, D.; Agnew, K.; Thomas, J.; Hitti, J. The relationship between racial identity, income, stress and C-reactive protein among parous women: implications for preterm birth disparity research. *Journal of the National Medical Association* **2008**, *100*, 540-546.
63. Pieczyńska, J.; Płaczkowska, S.; Pawlik-Sobecka, L.; Kokot, I.; Sozański, R.; Grajeta, H. Association of dietary inflammatory index with serum IL-6, IL-10, and CRP concentration during pregnancy. *Nutrients* **2020**, *12*, 2789.
64. Keenan‐Devlin, L.S.; Caplan, M.; Freedman, A.; Kuchta, K.; Grobman, W.; Buss, C.; Adam, E.K.; Entringer, S.; Miller, G.E.; Borders, A.E. Using principal component analysis to examine associations of early pregnancy inflammatory biomarker profiles and adverse birth outcomes. *American Journal of Reproductive Immunology* **2021**, *86*, e13497.
65. Ryu, H.K.; Moon, J.H.; Heo, H.J.; Kim, J.W.; Kim, Y.H. Maternal c‐reactive protein and oxidative stress markers as predictors of delivery latency in patients experiencing preterm premature rupture of membranes. *International Journal of Gynecology & Obstetrics* **2017**, *136*, 145-150.
66. Chul Sung, K.; Suh, J.Y.; Kim, B.S.; Kang, J.H.; Kim, H.; Lee, M.H.; Park, J.R.; Kim, S.W. High sensitivity C-reactive protein as an independent risk factor for essential hypertension. *American journal of hypertension* **2003**, *16*, 429-433.
67. Ali, M.; Hameed, B.; Kamel, W. The association of serum cancer antigen 125 and c–reactive protein level with the severity of preeclampsia. *Karbala J Med* **2012**, *5*, 1322-1328.
68. Seyhanli, Z.; Bayraktar, B.; Cakir, B.T.; Bucak, M.; Karabay, G.; Aktemur, G.; Yigit, A.; Yucel, K.Y.; Yılmaz, Z.V. The Efficacy of C‐Reactive Protein (CRP) to Albumin Ratio (CAR) and Fibrinogen to CRP Ratio (FCR) in Predicting the Latent Period of Preterm Labor. *American Journal of Reproductive Immunology* **2024**, *92*, e13899.

69. Shafiq, M.; Mathad, J.S.; Naik, S.; Alexander, M.; Yadana, S.; Araújo-Pereira, M.; Kulkarni, V.; Deshpande, P.; Kumar, N.P.; Babu, S. Association of maternal inflammation during pregnancy with birth outcomes and infant growth among women with or without HIV in India. *JAMA Network Open* **2021**, *4*, e2140584-e2140584.
70. Song, J.S.; Woo, S.J.; Park, K.H.; Kim, H.; Lee, K.-N.; Kim, Y.M. Association of inflammatory and angiogenic biomarkers in maternal plasma with retinopathy of prematurity in preterm infants. *Eye* **2023**, *37*, 1802-1809.
71. Suwardewa, T.G.A.; Sanjaya, I.N.H.; Anantasika, A.A.N.; Aryana, M.B.D.; Widiyanti, E.S.; Kurniawan, P.I. Correlation between group B Streptococcus infection in the vagina with maternal serum C-reactive protein levels in preterm labor. *European Journal of Medical and Health Sciences* **2022**, *4*, 18-21.
72. Yeates, A.J.; McSorley, E.M.; Mulhern, M.S.; Spence, T.; Crowe, W.; Grzesik, K.; Thurston, S.; Watson, G.; Myers, G.; Davidson, P. Associations between maternal inflammation during pregnancy and infant birth outcomes in the Seychelles Child Development Study. *Journal of reproductive immunology* **2020**, *137*, 102623.
