# Supplementary material for: Association Between Maternal C-Reactive Protein (CRP) Levels and Adverse Neonatal Outcomes: A Systematic Review and Meta-Analysis
Source: J Clin Med. 2026 Mar 10;15(6):2114. doi: 10.3390/jcm15062114 (PMC13027311; doi:10.3390/jcm15062114)
Supplement: Supplementary file 1 [file jcm-15-02114-s001.zip › CRP_Supplementary File S3.pdf]

Supplementary File S3. Sensitivity analysis of the association between maternal c-reactive protein (CRP) levels and adverse neonatal outcomes: **[A]** standardized mean difference (SMD) of maternal CRP levels in overall adverse neonatal outcomes compared with controls, **[B]** odds of preterm birth in relation to elevated maternal CRP, **[C]** odds of low birth weight in relation to elevated maternal CRP, **[D]** odds of small for gestational age in relation to maternal CRP, and **[E]** odds of stillbirth in relation to maternal CRP.

[A]

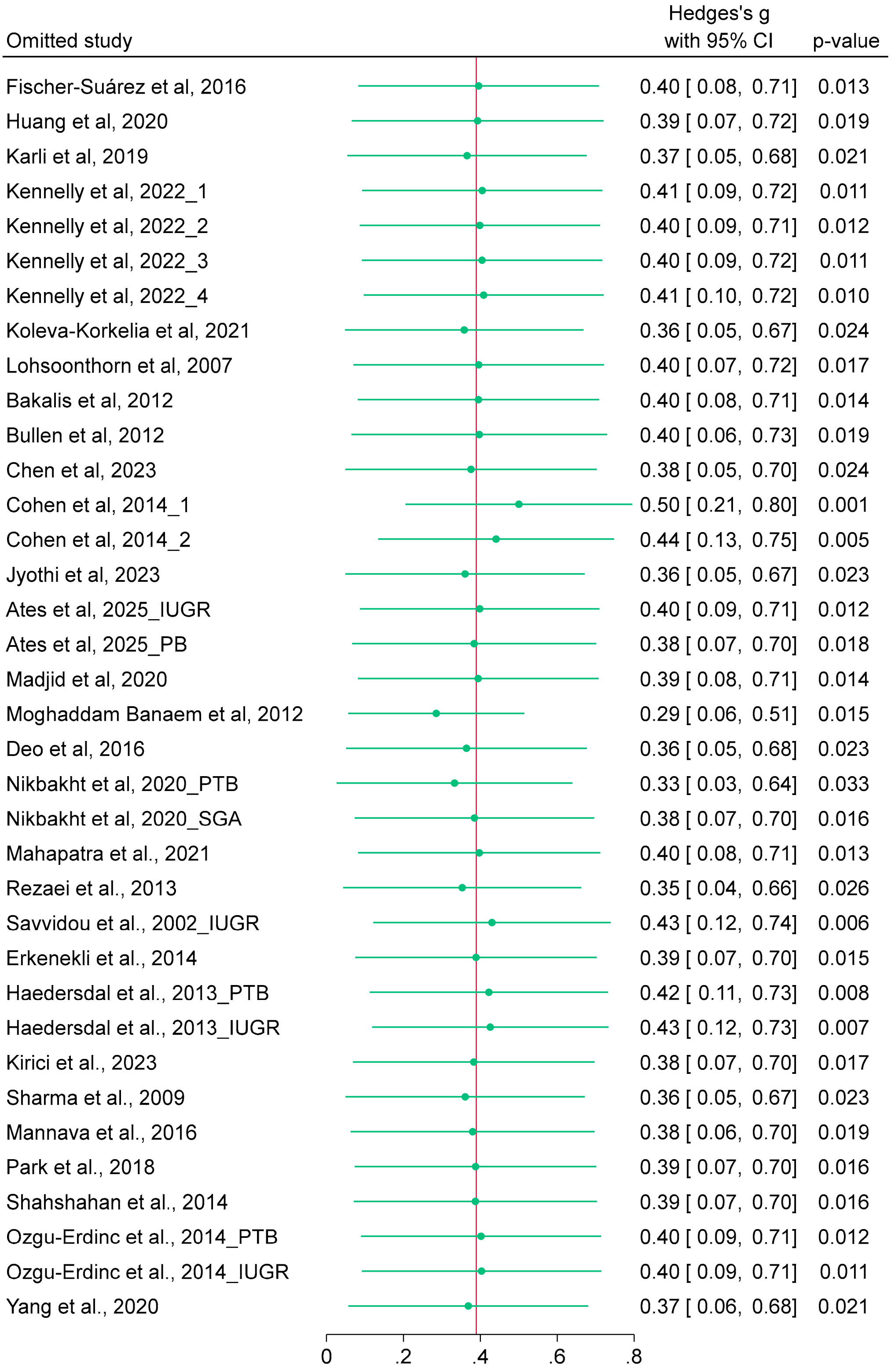

Random-effects DerSimonian–Laird model

[B]

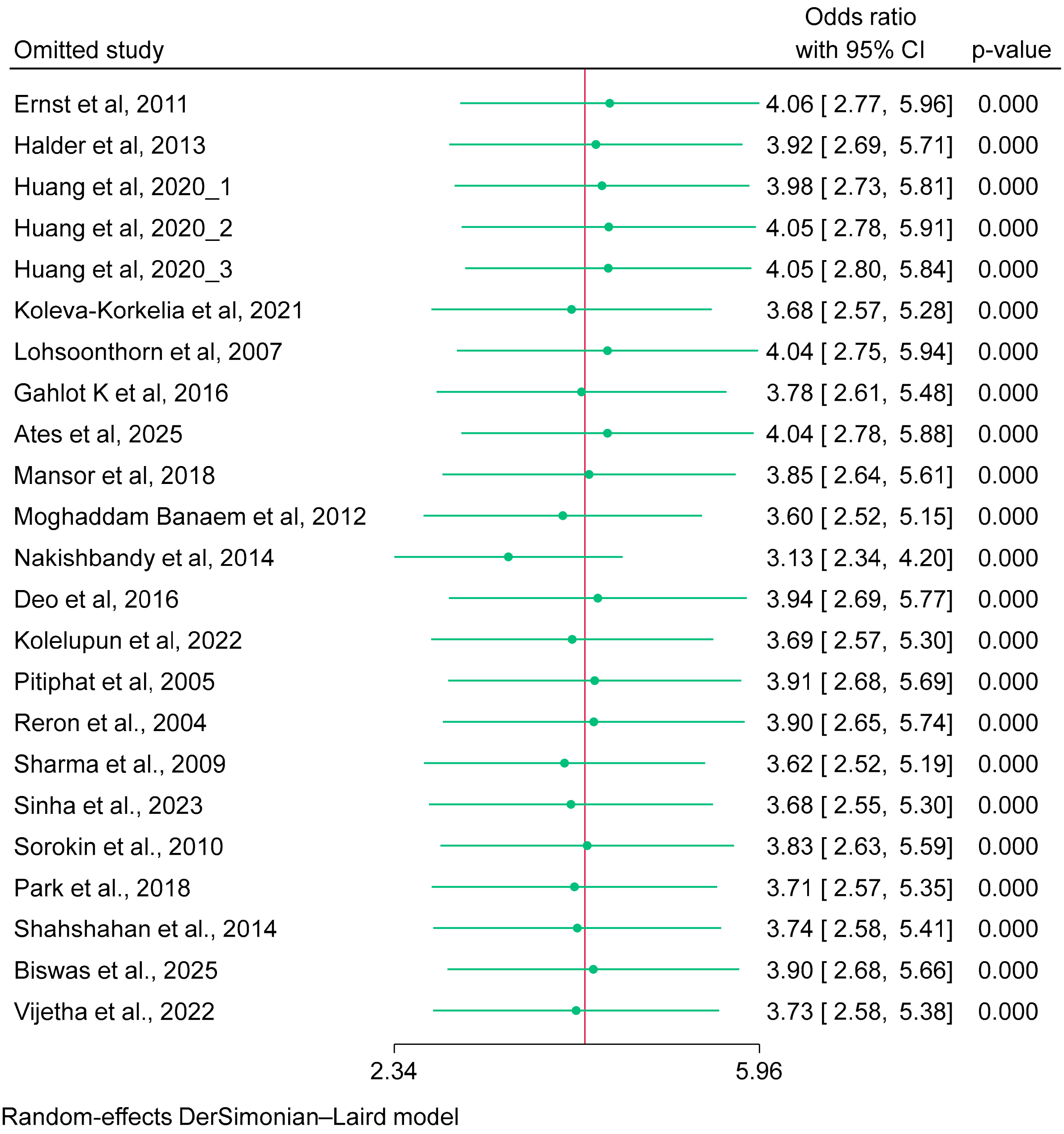
Omitted study
Odds ratio with 95% CI
p-value
Ernst et al, 2011 4.06 [2.77, 5.96] 0.000
Halder et al, 2013 3.92 [2.69, 5.71] 0.000
Huang et al, 2020_1 3.98 [2.73, 5.81] 0.000
Huang et al, 2020_2 4.05 [2.78, 5.91] 0.000
Huang et al, 2020_3 4.05 [2.80, 5.84] 0.000
Koleva-Korkelia et al, 2021 3.68 [2.57, 5.28] 0.000
Lohsoonthorn et al, 2007 4.04 [2.75, 5.94] 0.000
Gahlot K et al, 2016 3.78 [2.61, 5.48] 0.000
Ates et al, 2025 4.04 [2.78, 5.88] 0.000
Mansor et al, 2018 3.85 [2.64, 5.61] 0.000
Moghaddam Banaem et al, 2012 3.60 [2.52, 5.15] 0.000
Nakishbandy et al, 2014 3.13 [2.34, 4.20] 0.000
Deo et al, 2016 3.94 [2.69, 5.77] 0.000
Kolelupun et al, 2022 3.69 [2.57, 5.30] 0.000
Pitiphat et al, 2005 3.91 [2.68, 5.69] 0.000
Reron et al., 2004 3.90 [2.65, 5.74] 0.000
Sharma et al., 2009 3.62 [2.52, 5.19] 0.000
Sinha et al., 2023 3.68 [2.55, 5.30] 0.000
Sorokin et al., 2010 3.83 [2.63, 5.59] 0.000
Park et al., 2018 3.71 [2.57, 5.35] 0.000
Shahshahan et al., 2014 3.74 [2.58, 5.41] 0.000
Biswas et al., 2025 3.90 [2.68, 5.66] 0.000
Vijetha et al., 2022 3.73 [2.58, 5.38] 0.000
2.34
5.96
Random-effects DerSimonian–Laird model

[C]

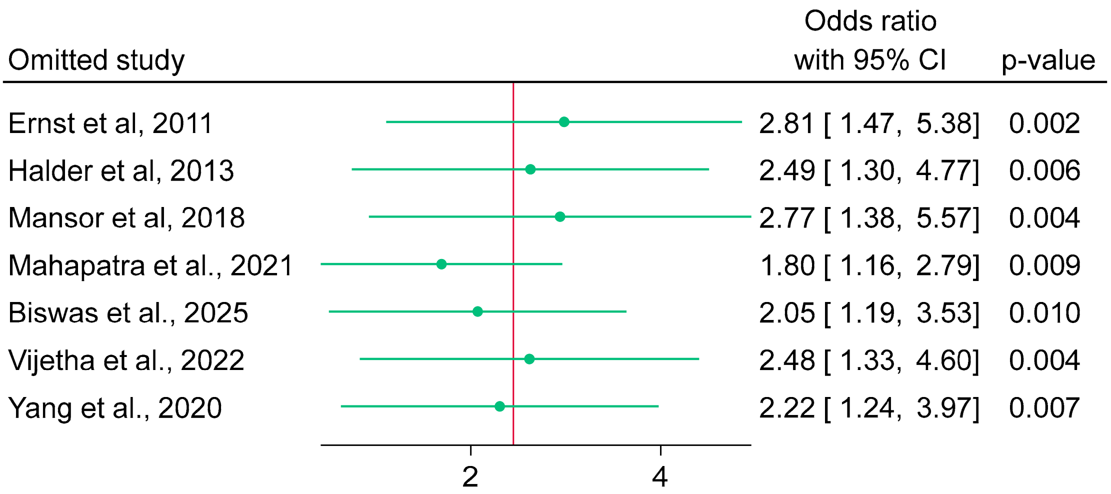

Random-effects DerSimonian–Laird model

[D]

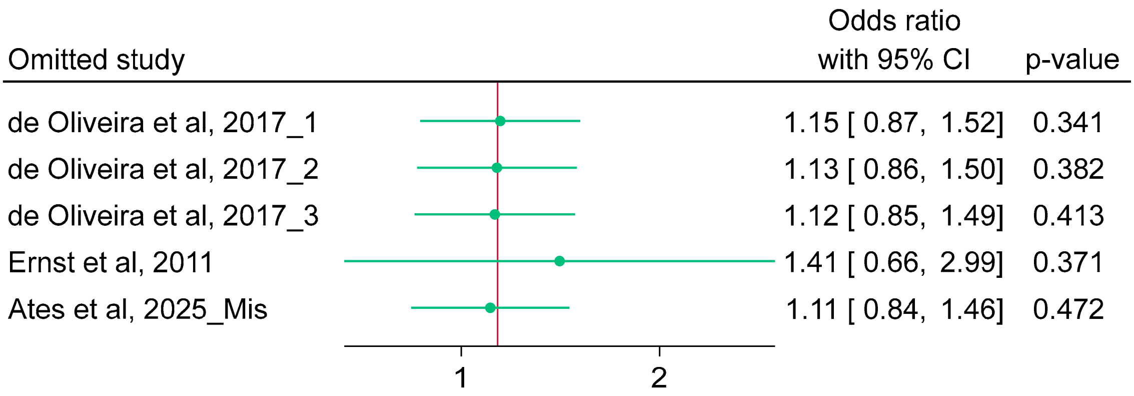

Random-effects DerSimonian–Laird model

[E]

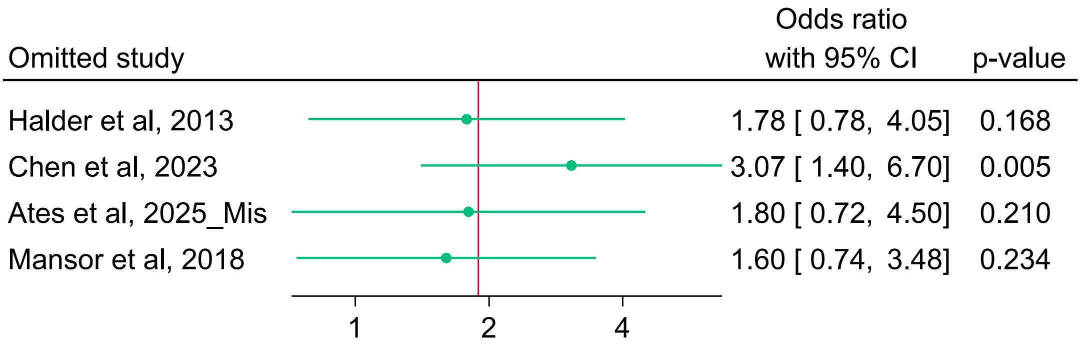

Random-effects DerSimonian–Laird model
