# Supplementary material for: Association Between Maternal C-Reactive Protein (CRP) Levels and Adverse Neonatal Outcomes: A Systematic Review and Meta-Analysis
Source: J Clin Med. 2026 Mar 10;15(6):2114. doi: 10.3390/jcm15062114 (PMC13027311; doi:10.3390/jcm15062114)
Supplement: Supplementary file 1 [file jcm-15-02114-s001.zip › CRP_Supplementary File S4.pdf]

Supplementary File S4. Funnel plots assessing publication bias for the association between maternal CRP levels and adverse neonatal outcomes, with Egger's and Begg's test p-values reported for each outcome: **[A]** standardized mean difference (SMD) of maternal CRP levels in overall adverse neonatal outcomes compared with controls, Egger's p = 0.022, Begg's p = 0.334, **[B]** odds of preterm birth in relation to elevated maternal CRP, Egger's p < 0.001, Begg's p < 0.001, **[C]** odds of low birth weight in relation to elevated maternal CRP, Egger's p < 0.001, Begg's p = 0.072, **[D]** odds of small for gestational age in relation to maternal CRP, Egger's p = 0.388, Begg's p = 0.221, and **[E]** odds of stillbirth in relation to maternal CRP.

[A]

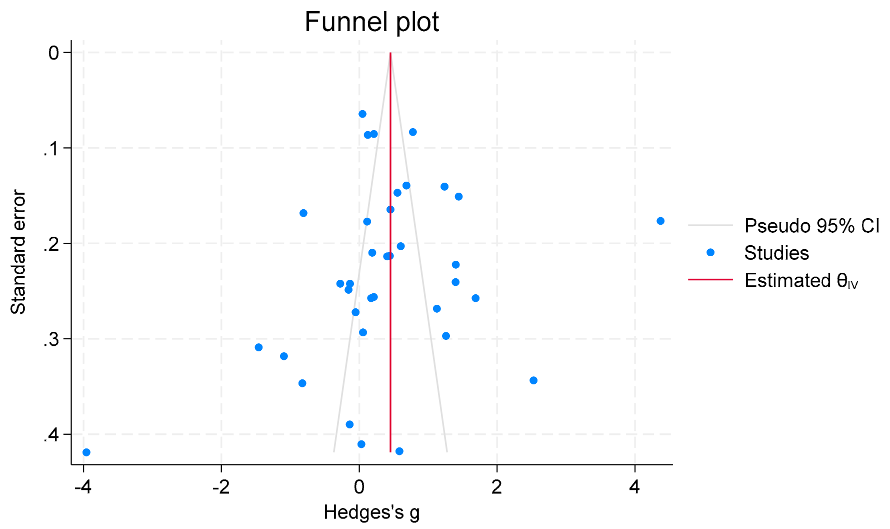

[B]

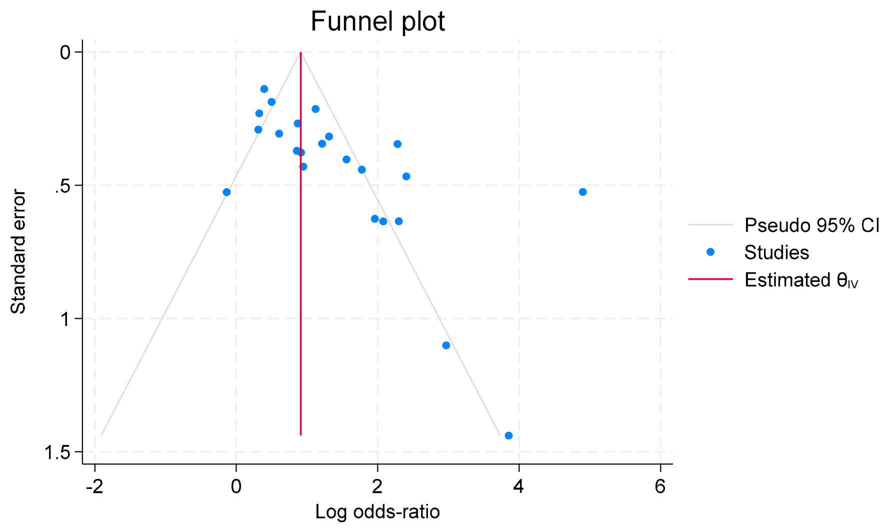

[C]

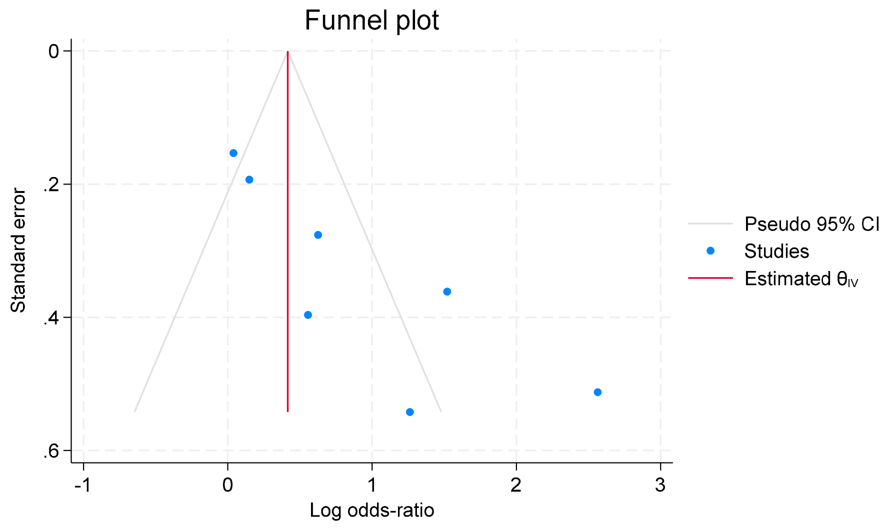
Funnel plot
Standard error
0
.2
.4
.6
-1
0
1
2
3
Log odds-ratio
Pseudo 95% CI
Studies
Estimated θIV

[D]

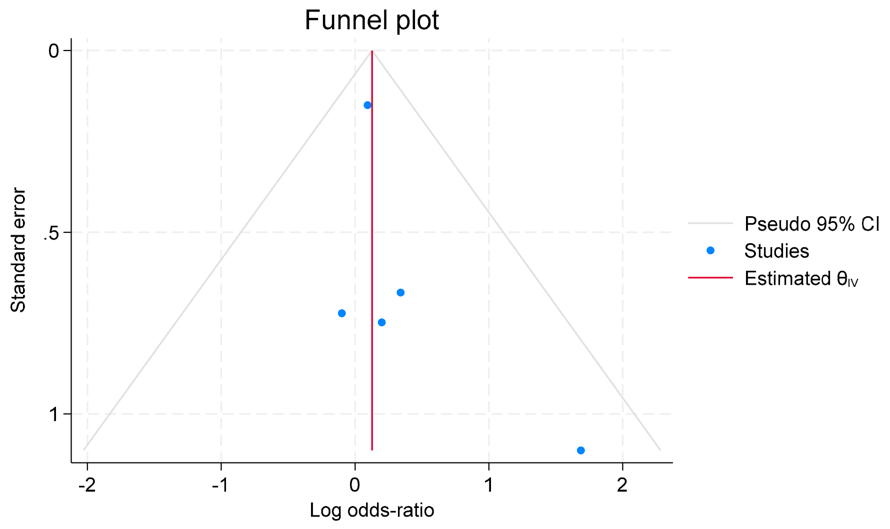
Funnel plot
Standard error
0
.5
1
-2
-1
0
1
2
Log odds-ratio
Pseudo 95% CI
Studies
Estimated θIV

[E]

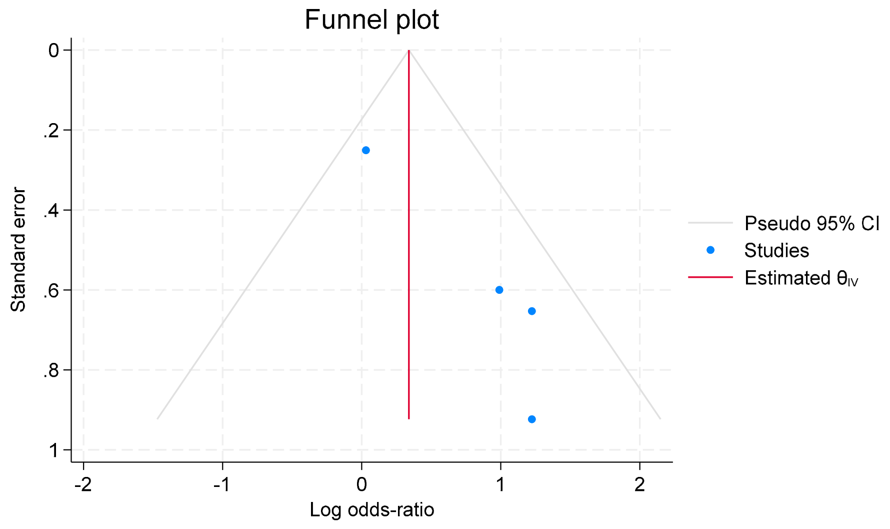
Funnel plot
Standard error
0
.2
.4
.6
.8
1
-2
-1
0
1
2
Log odds-ratio
Pseudo 95% CI
Studies
Estimated θIV
