# Supplementary material for: Association Between Maternal C-Reactive Protein (CRP) Levels and Adverse Neonatal Outcomes: A Systematic Review and Meta-Analysis
Source: J Clin Med. 2026 Mar 10;15(6):2114. doi: 10.3390/jcm15062114 (PMC13027311; doi:10.3390/jcm15062114)
Supplement: Supplementary file 1 [file jcm-15-02114-s001.zip › CRP_Supplementary File S5.pdf]

Supplementary File S5. Certainty of evidence based on GRADE approach for the analysis of the association between maternal CRP levels and adverse neonatal outcomes.

Certainty assessment							Certainty	Importance
№ of studies	Study design	Risk of bias	Inconsistency	Indirectness	Imprecision	Other considerations		
**Overall Neonatal complications**								
28	non-randomised studies	not serious	serious[a]	not serious	not serious	none	⊕⊕⊕◯ Moderate[a]	IMPORTANT
**Preterm Birth**								
21	non-randomised studies	not serious	serious[a]	not serious	not serious	none	⊕⊕⊕◯ Moderate[a]	IMPORTANT
**Low birth weight**								
7	non-randomised studies	not serious	serious[a]	not serious	not serious	none	⊕⊕⊕◯ Moderate[a]	IMPORTANT
**Small for gestational age**								
3	non-randomised studies	not serious	not serious	serious[b]	serious[c]	none	⊕⊕◯◯ Low[b,c]	IMPORTANT
**Stillbirth**								
4	non-randomised studies	not serious	not serious	serious[b]	serious[c]	none	⊕⊕◯◯ Low[b,c]	IMPORTANT

CI: confidence interval

Explanations

a. Serious Inconsistency since I^2 is more than 60%. Downgraded.
b. Serious Indirectness since limited number of studies included. Downgraded.
c. Serious Imprecision since pooled effect size have a wide CI. Downgraded.
